# Metabolic modulation to improve MSC expansion and therapeutic potential for articular cartilage repair

**DOI:** 10.1186/s13287-024-03923-w

**Published:** 2024-09-16

**Authors:** Ching Ann Tee, Daniel Ninio Roxby, Rashidah Othman, Vinitha Denslin, Kiesar Sideeq Bhat, Zheng Yang, Jongyoon Han, Lisa Tucker-Kellogg, Laurie A. Boyer

**Affiliations:** 1 Critical Analytics for Manufacturing Personalised-medicine (CAMP) Interdisciplinary Research Group, Singapore-MIT Alliance for Research and Technology (SMART) Centre, 1 Create Way, Enterprise Wing, #04-13/14, Singapore, 138602 Republic of Singapore; 2 NUS Tissue Engineering Program, Life Sciences Institute, National University of Singapore, 27 Medical Drive, DSO (Kent Ridge) Building, Level 4, Singapore, 117510 Republic of Singapore; 3 Department of Bioresources, University of Kashmir, Hazratbal, Srinagar, 190006 India; 4 Department of Orthopaedic Surgery, National University of Singapore, 1E Kent Ridge Road, NUHS Tower Block 11, Singapore, 119288 Republic of Singapore; 5 Cancer and Stem Cell Biology and Centre for Computational Biology, Duke-NUS Medical School, 8 College Rd, Singapore, 169857 Republic of Singapore; 6 Department of Electrical Engineering and Computer Science, Massachusetts Institute of Technology, 50 Vassar St, Cambridge, MA 02139 USA; 7 Department of Biological Engineering, Massachusetts Institute of Technology, 77 Massachusetts Avenue, Cambridge, MA 02139 USA; 8 Department of Biology, Massachusetts Institute of Technology, 77 Massachusetts Avenue, Cambridge, MA 02139 USA

**Keywords:** Mesenchymal stromal cells, Metabolic modulation, Articular cartilage, Cell expansion, In-process monitoring tools, Critical quality attributes

## Abstract

**Background:**

Articular cartilage degeneration can result from injury, age, or arthritis, causing significant joint pain and disability without surgical intervention. Currently, the only FDA cell-based therapy for articular cartilage injury is Autologous Chondrocyte Implantation (ACI); however, this procedure is costly, time-intensive, and requires multiple treatments. Mesenchymal stromal cells (MSCs) are an attractive alternative autologous therapy due to their availability and ability to robustly differentiate into chondrocytes for transplantation with good safety profiles. However, treatment outcomes are variable due to donor-to-donor variability as well as intrapopulation heterogeneity and unstandardized MSC manufacturing protocols. Process improvements that reduce cell heterogeneity while increasing donor cell numbers with improved chondrogenic potential during expansion culture are needed to realize the full potential of MSC therapy.

**Methods:**

In this study, we investigated the potential of MSC metabolic modulation during expansion to enhance their chondrogenic commitment by varying the nutrient composition, including glucose, pyruvate, glutamine, and ascorbic acid in culture media. We tested the effect of metabolic modulation in short-term (one passage) and long-term (up to seven passages). We measured metabolic state, cell size, population doubling time, and senescence and employed novel tools including micro-magnetic resonance relaxometry (µMRR) relaxation time (T_2_) to characterize the effects of AA on improved MSC expansion and chondrogenic potential.

**Results:**

Our data show that the addition of 1 mM L-ascorbic acid-2-phosphate (AA) to cultures for one passage during MSC expansion prior to initiation of differentiation improves chondrogenic differentiation. We further demonstrate that AA treatment reduced the proportion of senescent cells and cell heterogeneity also allowing for long-term expansion that led to a > 300-fold increase in yield of MSCs with enhanced chondrogenic potential compared to untreated cells. AA-treated MSCs with improved chondrogenic potential showed a robust shift in metabolic profile to OXPHOS and higher µMRR T_2_ values, identifying critical quality attributes that could be implemented in MSC manufacturing for articular cartilage repair.

**Conclusions:**

Our results suggest an improved MSC manufacturing process that can enhance chondrogenic potential by targeting MSC metabolism and integrating process analytic tools during expansion.

**Supplementary Information:**

The online version contains supplementary material available at 10.1186/s13287-024-03923-w.

## Background

Mesenchymal stromal cells (MSCs) hold great promise for tissue regeneration, especially as a cell therapy for articular cartilage repair due to their availability, scalable expansion, and multilineage differentiation capability. MSC implantation has shown success for articular cartilage repair with the generation of hyaline-like cartilage and improvement in joint function [1–4]. However, the inconsistent therapeutic efficacy of MSCs [5], likely due to donor-to-donor variation and intra-population heterogeneity during expansion [4, 6–8], limits their use in the clinic.

Donor-to-donor variation attributed to harvesting sites, age, gender and physiological status has been well documented [4, 6–11]. Moreover, large clinical-scale expansion of MSCs to obtain a therapeutically relevant number of cells for implantation further imposes intra-population heterogeneity morphologically and functionally in culture-expanded MSCs [6, 8, 12]. Within a MSC population, cells exhibit varying differentiation potential toward the chondrogenic, osteogenic or adipogenic lineages [8, 13]. Moreover, upon expansion, MSC subpopulations display heterogeneous cell size distributions with varying differentiation potential [14–16]. Current MSC manufacturing protocols are not sufficient to overcome challenges of heterogeneity, which can be further impacted by the lack of standardization, insufficient monitoring, or suboptimal culture conditions. Stem cell metabolic plasticity in response to changing environment and differentiation state is well known [17], however, it is unclear whether metabolic manipulation of MSC during expansion culture could improve cell quality.

MSCs are sensitive to their microenvironment, and efforts have focused on priming MSCs toward specific cell types during differentiation by imposing physical, chemical, or biological cues. For example, L-ascorbic acid-2-phosphate (AA) is commonly supplemented in chondrogenic induction media as a cofactor of collagen prolyl hydroxylases to regulate collagen homeostasis [18–20]. For the same reason, AA is used in MSC cell sheet formation for cartilage engineering [21] and wound healing [22–24]. Although supplementation of AA to MSC culture media during expansion is not a common practice, some studies demonstrated a beneficial effect of AA treatment on MSC proliferation [25] and a decrease in reactive oxygen species (ROS) [26, 27]. However, systematic studies on AA supplementation during MSC expansion across multiple donors are lacking in the field.

## Methods

### Bone marrow derived MSC culture and expansion

Human bone marrow derived MSCs (Lonza and STEMCELL Technologies) from 4 donors (Additional File 1: Table S1) were characterized by immunophenotypic cell surface markers endorsed by the International Society of Cell and Gene Therapy (ISCT) [28] (Additional File 1: Figure S1 and Table S2) and expanded in standard expansion media composed of 1 g/L D-Glucose Dulbecco’s modified Eagle’s Medium (DMEM; Gibco), 10% fetal bovine serum (FBS; Gibco) and 1% (v/v) penicillin-streptomycin (Gibco). In the experimental groups, 0.05, 0.2 or 1.0 mM of L-ascorbic acid-2-phosphate (AA; Sigma-Aldrich; A8960) was added to the standard expansion media. Media was changed every 2–3 days. MSCs were seeded at 2000 cells/cm^2^ and maintained in a humidified 37 °C incubator with 5% CO_2_. MSCs were harvested with 0.05% Trypsin/EDTA solution (Gibco). Cell count and viability were determined using a hemocytometer (INCYTO) and Trypan Blue (Thermo Fisher).

For experiments on varying glucose, pyruvate, and glutamine concentrations, MSCs frozen at P1 were thawed and cultured until passage 3 (P3). At P3, MSCs were seeded at 2000 cells/cm^2^ and cultured in DMEM without glucose, glutamine, and sodium pyruvate (Gibco) with manual supplementation of standard glucose (5.5 mM; Gl5.5; Gibco), sodium pyruvate (1.0 mM; Py1.0; Gibco) and glutamine (4.0 mM; Gn4.0; Gibco) concentrations that served as Control. In experimental groups, 10x lower or 2x higher than standard concentrations were used, namely Gl0.5 (0.5 mM glucose), Gl11 (11 mM glucose), Py0.1 (0.1 mM pyruvate), Py2.5 (2.5 mM pyruvate), Gn0.5 (0.5 mM glutamine), and Gn8.0 (8.0 mM glutamine). After 7 days, MSCs were harvested for analysis of metabolic profiles and chondrogenic potential.

### Trilineage differentiation, histology, protein quantification

Chondrogenic differentiation was performed in cell pellets containing 1.5 × 10^5^ cells/pellet. The cell pellets were cultured in a chondrogenic medium composed of 4.5 g/L D-Glucose DMEM (Gibco) supplemented with 0.1 µM dexamethasone (Sigma-Aldrich), 50 µg/mL ascorbic acid (Sigma-Aldrich), 4 mM proline (Sigma-Aldrich), 1% ITS Premix supplement (Becton-Dickinson), 1 mM sodium pyruvate, 1% penicillin-streptomycin, 1 mM GlutaMax and 10 ng/ml TGF-β3 (RnD system). Chondrogenic differentiation media was changed every 2 days for 3 weeks before the pellets were harvested for histology and protein quantitative analysis. For histology, pellets were fixed in 10% neutral buffered formalin (Sigma-Aldrich) overnight at 4 °C before being dehydrated with graded ethanol and xylene. Dehydrated pellets were then embedded in paraffin blocks and sectioned into 5 μm thickness, followed by mounting on polylysine-coated slides. Cartilage matrix proteoglycan was identified by staining with 0.1% Safranin O solution (Acros Organics) and counterstained with 0.1% fast green and hematoxylin (Sigma-Aldrich). Type II Collagen (Col2) was identified by immunostaining with an Ultra Vision detection kit (ThermoFisher) and Col2 antibody (Clone 6B3 at 1:500 dilution; Chemicon), followed by biotinylated goat anti-mouse secondary antibody with horseradish peroxidase-streptavidin (Lab Vision). Quantification of sulfated glycosaminoglycan (sGAG), Col2 and DNA was performed with Blyscan™ sGAG assay kit (Biocolor), Type II Collagen Detection Kit (Chondrex), and Picogreen dsDNA assay (Molecular Probes), respectively, according to manufacturer’s protocol following pellet digestion. Pellets were digested with 1 mg/ml of pepsin solution in 0.05 M acetic acid, followed by 1 mg/ml elastase solution. sGAG and Col2 content were normalized to the DNA of the respective samples.

Osteogenic differentiation was performed in a 24-well plate at a cell density of 3 × 10^4^ cells per well in osteogenic differentiation media composed of 1 g/L D-Glucose DMEM supplemented with 0.5% FBS, 50 µg/ml ascorbic acid, 1 mM sodium pyruvate, 10 µM dexamethasone and 10 mM β-glycerophosphate. Osteogenic induction was confirmed with Alizarin Red S staining (ScienCell) for calcium deposits. The dye was extracted for optical density measurement at 405 nm using a plate reader (Tecan).

Adipogenic differentiation was performed in a 24-well plate at a cell density of 6 × 10^4^ cells per well in adipogenic differentiation media composed of 1 g/L D-Glucose DMEM supplemented with 10% FBS, 10 µg/ml insulin (Sigma-Aldrich), 0.5 mM isobutylmethylxanthine (Sigma-Aldrich), 10 µM dexamethasone and 200 µM indomethacin. Adipogenic induction was confirmed with Oil Red O staining (ScienCell) for lipid droplets. The dye was extracted for optical densities measurement at 510 nm using a plate reader.

### Metabolic assays

Oxygen consumption rate (OCR) associated with oxidative phosphorylation (OXPHOS) and extracellular acidification rate (ECAR) associated with secretion of lactic acid as a metabolic product of glycolysis were quantified using Seahorse XF24 Extracellular Flux Analyzer (Agilent Technologies). MSCs were seeded on a Seahorse 24-well microplate at 2000 cells/cm^2^ and expanded with standard expansion media with or without AA. On the day of analysis, cells were washed twice with Seahorse assay medium and kept in a 37 °C non-CO_2_ incubator prior to running the glycolysis stress test according to the manufacturer’s protocol. Three independent readings were taken after each sequential injection of glucose, oligomycin and 2DG with final concentrations of 10 mM, 1 µM and 50 mM, respectively. Data were analyzed using Wave software (Agilent Technologies). OCR and ECAR at the basal level were calculated by subtracting the reading after 2DG injection from the reading after glucose injection and normalized to cell number. OCR: ECAR ratio was calculated by dividing the basal OCR by basal ECAR.

Metabolites such as glucose and lactate concentrations in spent media were measured using Cedex Bio Analyze (Roche). Glucose consumption and lactate production rates were calculated by deducting the concentrations in spent media from the concentrations in fresh media and normalizing to the time of incubation and cell number.

### Micro-magnetic resonance relaxometry (µMRR) measurement

The setup of the µMRR instrument used in this study was described elsewhere [29]. For µMRR measurement, 3 × 10^5^ cells were suspended in 20 µL of PBS, and 4 µL of cell suspensions were loaded to the bottom 4 mm of micro-capillary tubes. The micro-capillary tubes were then sealed with critoseal (Leica Microsystems) and placed into the coil of µMRR instrument. Proton transverse relaxation time (T_2_) was measured by the standard Carr-Purcell-Meiboom-Gill (CPMG) pulse program.

### Suspended cell diameter measurement

MSC suspension was loaded to a hemacytometer chip, and images were taken at 10x magnification. Images were analyzed with MATLAB (The MathWorks Inc.) using an imaging-based MATLAB algorithm to obtain suspended cell diameters. Objects with measured diameters smaller than 10 μm and bigger than 75 μm were excluded from the analysis. At least 500 cells were captured and analyzed in each group.

### Identification of MSC surface markers by flow cytometry

Harvested cells were washed once with phosphate-buffered saline (PBS) and stained with fluorochrome-conjugated anti-human antibodies: CD73-fluorescein isothiocyanate (FITC), CD90-phycoerythrin (PE), CD105-allophycocyanin (APC), CD34-Brilliant Violet (BV) 510 and CD45-peridinin chlorophyll protein (PerCP) Cy5.5 (BD Biosciences). The acquisition was performed using a CytoFLEX flow cytometer (Beckman Coulter) and analysed using FlowJo V10. Positively stained cell populations were identified by comparing them with fluorescence-minus-one (FMO) controls.

### Beta-galactose staining

Harvested MSCs were seeded at 5000 cells/cm^2^ on a 6-well plate. On the next day, senescent cells were identified using a senescence β-galactosidase staining kit (Sigma-Aldrich) according to the manufacturer’s protocol. Briefly, the cells were fixed at room temperature for 10 min, followed by incubation in a staining solution in a 37 °C non-CO_2_ incubator overnight. After which, 5 random areas in each well were captured, and the percentage of positively stained cells was calculated.

### Statistical analysis

Data were presented as mean ± standard deviation. Statistical analysis between the two groups were carried out by Student’s t-test using the Microsoft Excel software with *p* < 0.05 indicating statistical significance.

## Results

### AA treatment during expansion specifically improves the chondrogenic potential of MSCs

Standard MSC expansion media contains physiological concentrations of glucose, pyruvate, and glutamine, which are 5.5 mM (Gl5.5), 1.0 mM (Py1.0) and 4.0 mM (Gn4.0), respectively. Donor 1 MSCs (Additional File 1: Figure S1, Tables S1 and S2) were seeded at 2000 cells/cm^2^ at passage 3 (P3) and expanded in media with varying glucose, pyruvate, and glutamine concentrations for 7 days prior to harvesting at P4, the time point commonly used for implantation. We observed that culturing MSCs at 10-fold lower standard glucose (Gl0.5) or pyruvate (Py0.1) concentrations improved MSC chondrogenic potential (Additional File 1: Figure S2A) as indicated by higher sGAG production compared to standard concentrations, while no difference was observed in varying glutamine concentrations (Additional File 1: Figure S2A). Moreover, Gl0.5 and Py0.1 conditions led to a metabolic shift toward oxidative phosphorylation (OXPHOS) as shown by a lower lactate production rate to glucose consumption rate ratio and higher oxygen consumption rate (OCR) during basal respiration compared to standard (Gl5.5 or Py1.0). The experiment was repeated for Donors 2 and 3 (Additional File 1: Figure S1, Tables S1 and S2). However, the beneficial effect of Gl0.5 and Py0.1 on chondrogenesis was highly donor-dependent (Additional File 1: Figure S2B). Nevertheless, these data showed that the ratio of total sGAG to total DNA is negatively correlated with glycolysis and positively correlated with OXPHOS (Additional File 1: Figure S3). Our results suggest that metabolic manipulation during MSC culture could be adopted to prime the chondrogenic potential.

**Fig. 1:**
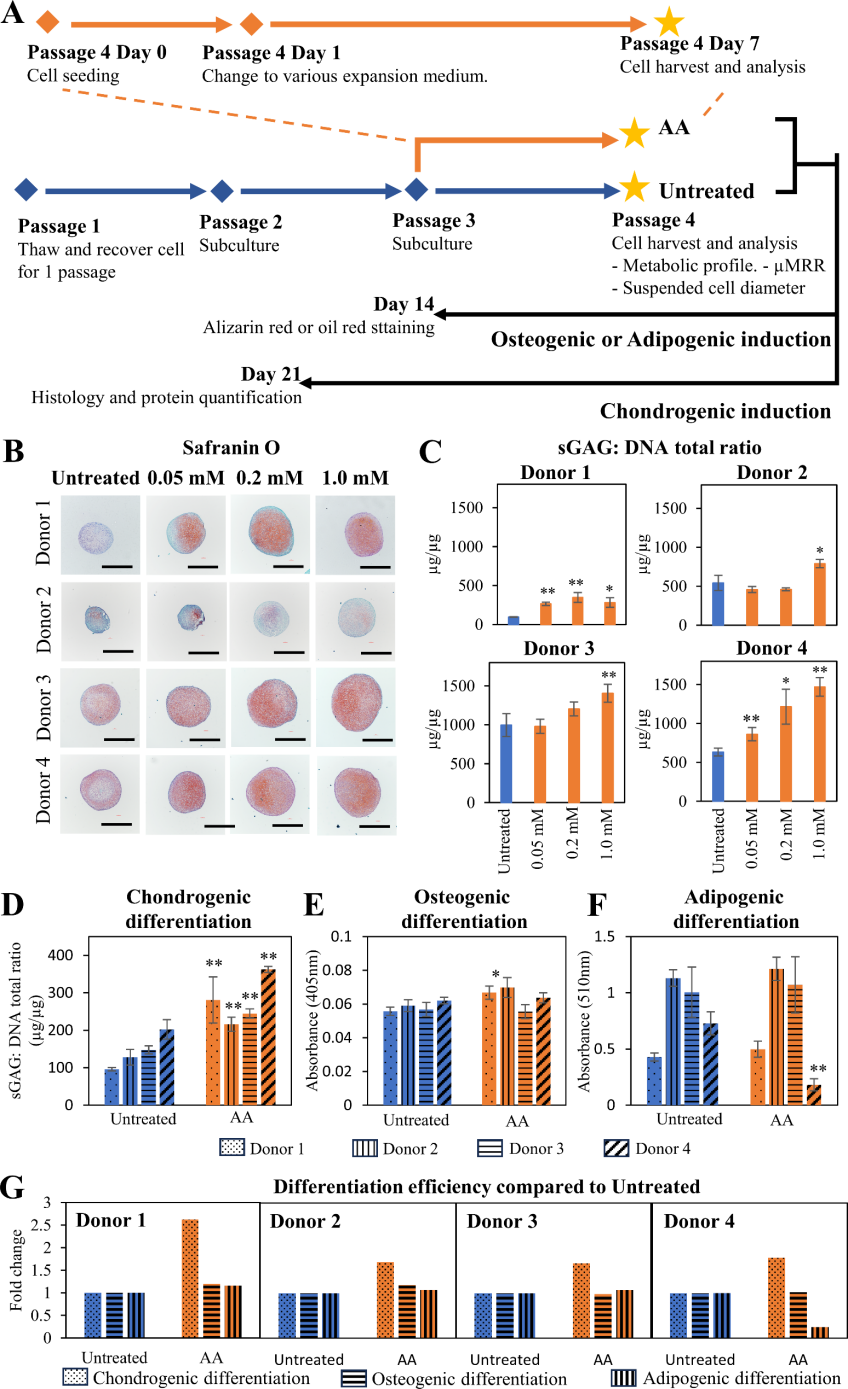
Effect of AA treatment in MSC priming during expansion on subsequent chondrogenic differentiation. **(A)** Schematic diagram of the experiment setup. MSCs frozen at P1 were recovered and sub-cultured until passage 3. Passage 3 MSCs were seeded and allowed to adhere overnight before the media was changed to expansion media containing 0 (Untreated), 0.05, 0.2 or 1.0 mM AA. MSCs were harvested at Passage 3 on Day 7 for subsequent analysis. **(B)** Histology of chondrogenic pellets after 3 weeks of chondrogenic differentiation following 1 passage of 0 (Untreated), 0.05, 0.2 and 1.0 mM of AA treatment. Formation of glycosaminoglycan (sGAG) indicated by Safranin O staining. 40x magnification; scale bar: 500 μm. Images are representative of 5 replicates per donor. **(C)** Quantification of sGAG in digested pellets normalized to total DNA per pellet. (D, E, F) Trilineage differentiation capacity of MSCs following 1 passage of 0 (Untreated) or 1.0mM AA (AA) treatment. **(D)** Chondrogenic differentiation is indicated by the ratio of total sGAG to total DNA in chondrogenic pellet; **(E)** osteogenic differentiation is represented by the amount of Alizarin red stain for calcium deposits, and **(F)** adipogenic differentiation is represented by the amount of oil red stain for oil droplets. **(G)** Comparison between chondrogenic, osteogenic and adipogenic differentiation efficiency in relative to Untreated following 1 passage of 0 (Untreated) or 1.0 mM AA (AA) treatment. Experiments were performed in 3–4 technical replicates. Data are presented as mean ± standard deviation. * *P* < 0.05 and ** *P* < 0.01 compared to Untreated

Given that AA is reported to support OXPHOS [30] and supplementation during MSC differentiation leads to improved chondrogenesis [18–20], we investigated the effect of AA supplementation during MSC expansion. To this end, we supplemented AA at P2 and P3 prior to harvesting cells for analysis at P4. We observed similar results comparing 1 and 2 passages of AA treatment (data not shown). Thus, for our studies, we treated MSCs from 4 characterized donors (Additional File 1: Figure S1, Table S1 and S2) with varying concentrations of AA (0.05, 0.2 and 1.0 mM) at P3 for 7 days prior to harvesting at P4 for chondrogenic differentiation (Fig. 1A). MSCs in standard expansion media served as the control (Untreated). Upon harvesting expanded MSCs, chondrogenic differentiation in 3D pellets was induced for 21 days [14, 31–34]. Histological analysis and protein quantification of chondrogenic pellets showed that AA treatment improved MSC chondrogenic potential based on enhanced production of articular cartilage matrix, sGAGs (Fig. 1B and C) and Col2 (Additional File 1: Figures S4A and S4B). The improvement observed in AA treated group was further supported by higher expression of the chondrogenic marker *SOX9* (Additional File 1: Figures S4C), a master transcription factor that plays an essential role during early phases of chondrogenic differentiation [35, 36]. Although we observed variable dose-response profiles among donors (Fig. 1C and Additional File 1: Figure S4B), significantly increased chondrogenic potential was consistently observed at 1.0 mM AA across all 4 donors, so we used this concentration in subsequent experiments.

We next measured the effect of AA treatment on osteogenic and adipogenic differentiation. AA treatment during MSC expansion improved chondrogenic (Fig. 1D) differentiation, while no differences were observed in osteogenic and adipogenic differentiation (Fig. 1E and F), compared to untreated controls across all 4 Donors. By comparing chondrogenic, osteogenic, and adipogenic differentiation relative to Untreated using standard markers (Fig. 1G), AA supplementation during expansion showed specific improvement for chondrogenic differentiation. We also analyzed the immunophenotypic cell surface markers endorsed by the ISCT [28]. Results from flow cytometry showed that both Untreated and AA-treated MSCs displayed positive staining for CD73, CD90 and CD105 and minimal staining for CD34 and CD45 after expansion, suggesting AA treatment did not alter overall MSC quality (Additional File 1: Figure S5 and Table S3).

### AA treatment shifts MSC metabolism toward the OXPHOS phenotype

**Fig. 2:**
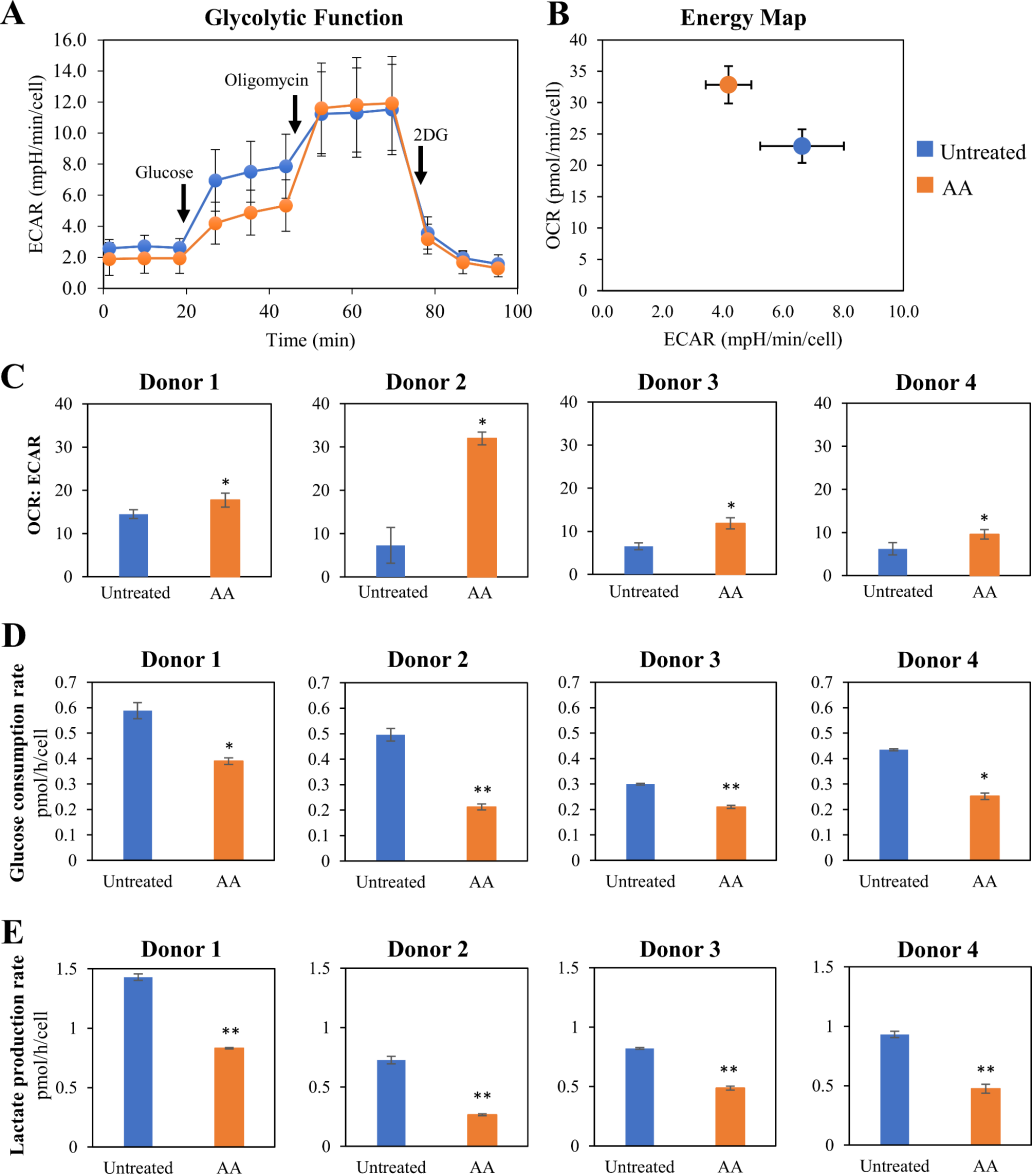
Effect of AA treatment in MSC priming during expansion on MSC metabolic profile. **(A)** MSC glycolytic function represented by real-time extracellular acidification rate (ECAR) plot of Untreated and AA-treated MSCs from Donor 1 in response to glucose, oligomycin and 2-deoxyglucose (2-DG) in Seahorse XF Glycolysis Stress test. **(B)** Energy map of Untreated and AA-treated MSCs presenting both ECAR and oxygen consumption rate (OCR) at the basal level. **(C)** OCR: ECAR ratio of Untreated and AA-treated MSCs at the basal level. **(D)** Glucose consumption and **(E)** lactate production rates were measured from the changes in the glucose and lactate concentrations in fresh and spent media, normalized to the total number of cells and hours of incubation. Experiments were performed in 3 technical replicates. Data are presented as mean ± standard deviation. * *P* < 0.05 and ** *P* < 0.01 compared to Untreated

Our data show that MSC chondrogenic potential is negatively correlated with glycolysis and positively correlated with OXPHOS (Additional File 1: Figure S3). Thus, we investigated the metabolic state of MSCs following 7 days of AA treatment. Using a Seahorse flux analyzer (Fig. 2A), we monitored both the OCR, an indicator for OXPHOS and ECAR, an indicator of glycolytic flux. At the basal level, AA-treated MSCs had higher OCR and lower ECAR (higher OCR: ECAR ratio) indicating increased OXPHOS compared to Untreated MSCs (Fig. 2B and C). The decreased glycolytic profile in AA-treated MSCs was further supported by lower glucose consumption (Fig. 2D) and lactate production rates (Fig. 2E) measured in spent media. Our results show that AA supplementation at P3 for 7 days prior to harvesting at P4 led to a shift toward an OXPHOS metabolic state and improved chondrogenic potential.

### AA-treated MSCs display differential µMRR T_2_ values and suspended cell diameter

**Fig. 3:**
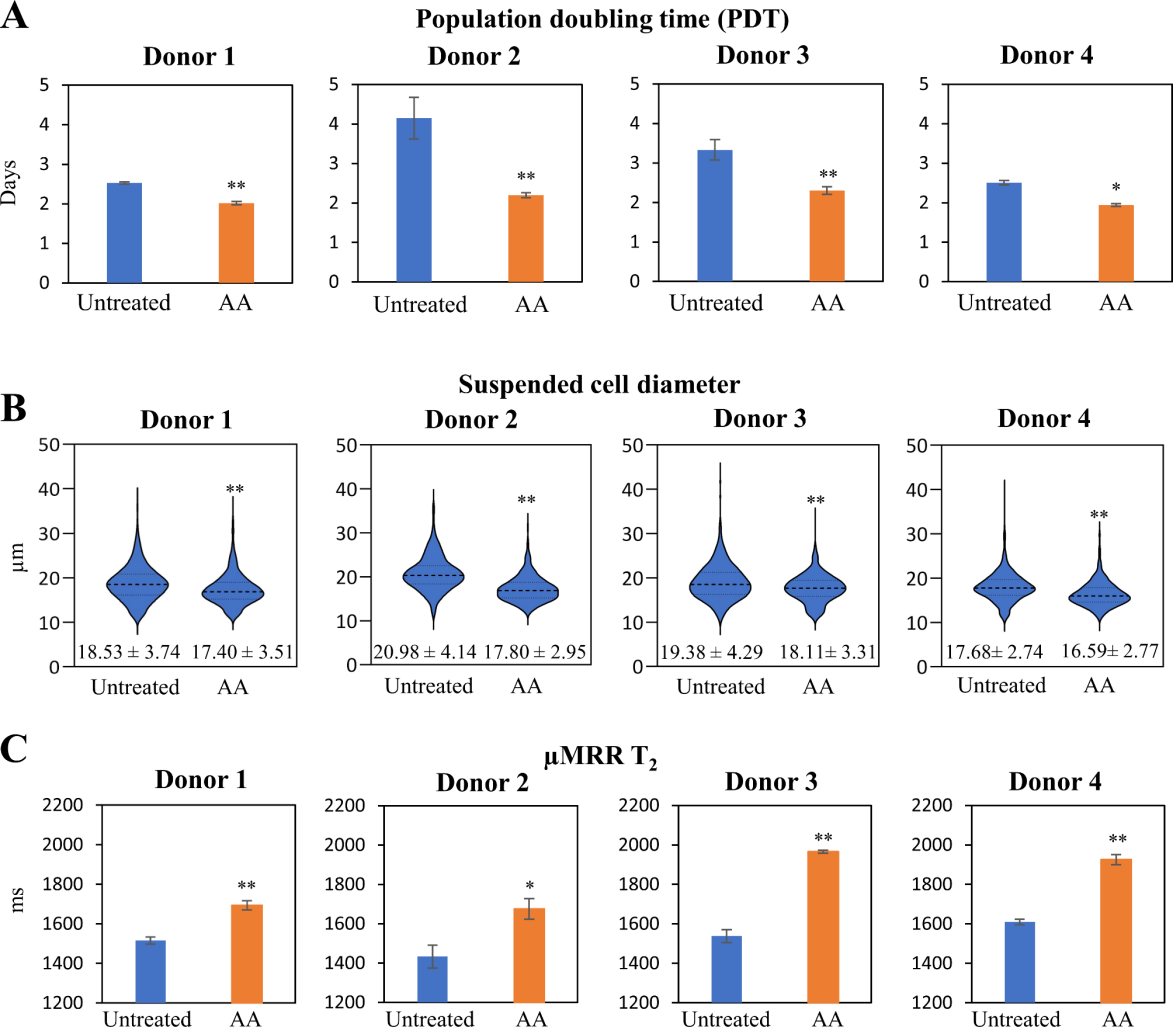
Effect of AA treatment on MSC proliferation and potential critical quality attributes. **(A)** Population doubling time (PDT) of Untreated and AA-treated MSCs at Passage 4. 7 days of 1 mM AA treatment led to a reduction and highly similar PDT across 4 donors (Donor 1: 2.04 ± 0.035 days; Donor 2: 1.94 ± 0.034 days; Donor 3: 2.30 ± 0.096 days; Donor 4: 2.20 ± 0.061 days), compared to Untreated MSCs (Donor 1: 2.53 ± 0.026 days; Donor 2: 4.15 ± 0.530 days; Donor 3: 3.33 ± 0.260 days; Donor 4: 2.51 ± 0.054 days) that showed heterogeneity in PDT as measured by final cell yield at P4 with cell counting using Trypan blue. From initial cell seeding density of 0.2 × 10^4^ cells/cm^2^, AA treatment resulted in higher cell yield (Donor 1: 1.69 ± 0.05 × 10^4^ cells/cm^2^; Donor 2: 1.82 ± 0.08 × 10^4^ cells/cm^2^; Donor 3: 1.64 ± 0.14 × 10^4^ cells/cm^2^; Donor 4: 2.41 ± 0.10 × 10^4^ cells/cm^2^) as compared to Untreated MSCs (Donor 1: 1.01 ± 0.01 × 10^4^ cells/cm^2^; Donor 2: 0.66 ± 0.10 × 10^4^ cells/cm^2^; Donor 3: 0.86 ± 0.10 × 10^4^ cells/cm^2^; Donor 4: 1.39 ± 0.06 × 10^4^ cells/cm^2^). **(B)** Suspended cell diameter of Untreated and AA-treated MSCs. Measurements were calculated from 400–500 cells. Data are presented in violin plots with the first dotted line as the 75th percentile; the second dotted line as the mean and the last dotted line as the 25th percentile; the value below each violin plots as mean ± standard deviation. **(C)** Micro-magnetic Resonance Relaxometry (µMRR) T_2_ relaxation time of Untreated and AA-treated MSCs. µMRR measurements were performed in 3 technical replicates. Data are presented as mean ± standard deviation. * *P* < 0.05 and ** *P* < 0.01 compared to Untreated

AA treatment has been reported to maintain MSC proliferation in culture [25]. Consistent with prior studies, we find that 7 days of 1 mM AA treatment led to a reduction and highly similar population doubling time (PDT) across 4 donors, compared to Untreated MSCs that showed heterogeneity in PDT as measured by final cell yield at P4 with cell counting using Trypan blue (Fig. 3A and Additional File 1: Table S4). MSC proliferation following AA treatment has been attributed to its antioxidant activity, which may reduce the proportion of senescent cells during expansion [27]. One hallmark of senescent cells is enlarged cell size [37, 38]. In prior work, MSCs with cell diameters larger than 23 μm correlated with cellular senescence [14, 29], while MSCs with a size range between 17 and 21 μm displayed better chondrogenic potential [14, 15]. Notably, we observed a significantly smaller and more homogeneous suspended cell diameter in AA-treated MSCs compared to Untreated controls (Fig. 3B and Additional File 1: Figure S6).

Emerging evidence also indicates that senescent cells accumulate Fe^3+^ due to alterations in iron homeostasis, inhibiting ferritinophagy [39, 40]. µMRR has been employed as a sensitive method for measuring differences in intracellular paramagnetic iron concentration [29]. For example, MSCs treated with senescent agents or over long-term passage reproducibly showed a lower µMRR T_2_ relaxation time that correlates with increased iron and potentially increased reactive oxygen species [29]. T_2_ values also correlated with MSC chondrogenic potential, while no correlation was observed for osteogenic or adipogenic differentiation [29]. Remarkably, we found that AA-treated MSCs harvested at P4 with improved proliferation and chondrogenic potential showed a significantly higher µMRR T_2_ relaxation time compared to untreated MSCs across all 4 donors (Fig. 3C). These data suggest that AA supplementation maintained iron homeostasis and reduced oxidative stress, as also supported by a marked reduction in population doubling and in the proportion of senescent cells. Our results support the potential of µMRR as a process monitoring tool for the expansion of MSCs for cell therapy.

### Long-term AA supplementation during MSC expansion improves cell yield and chondrogenic potential

**Fig. 4:**
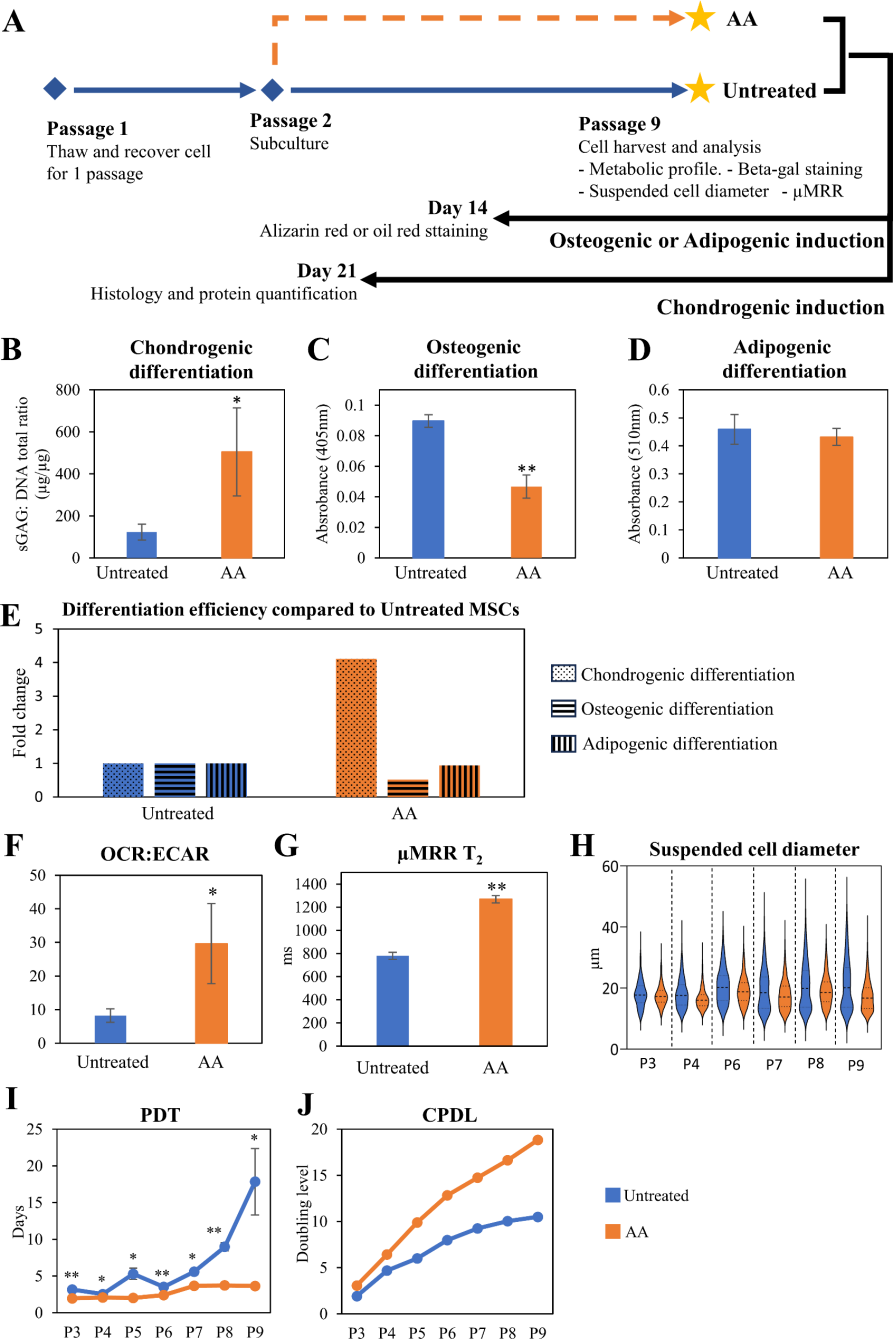
Effect of long-term AA supplementation in MSC manufacturing. **(A)** Schematic diagram of the experiment setup. MSCs frozen at P1 were thawed and recovered for 1 passage in standard expansion media. From passage 2 to 9, AA was supplemented with standard expansion media in the experimental group (AA), whereas the Untreated control was cultured in standard expansion media without AA. MSCs were harvested at passage 9 for subsequent analysis. **(B)** Chondrogenic differentiation is indicated by the ratio of total sGAG to total DNA in chondrogenic pellets; **(C)** osteogenic differentiation is represented by the amount of Alizarin red stain for calcium deposits; and **(D)** adipogenic differentiation is represented by the amount of oil red stain for oil droplets. **(E)** Comparison between chondrogenic, osteogenic and adipogenic differentiation efficiency of AA-treated MSCs in relative to Untreated. **(F)** Metabolic profile of Untreated and AA treated MSCs, presented as OCR: ECAR at passage 9 (P9) **(G)** µMRR T_2_ relaxation time of Untreated and AA treated MSCs at P9. **(H)** Suspended cell diameter of Untreated and AA-treated MSCs from P3 to P9. Measurements were calculated from 400–500 cells. Data are presented in violin plots with the first dotted line as the 75th percentile, the second dotted line as the mean and the last dotted line as the 25th percentile. **(I)** Population doubling time (PDT) and **(J)** cumulative population doubling level (CPDL) of Untreated and AA-treated MSCs from P3 to P9. With an initial cell seeding density of 2.0 × 10^3^ cells/cm^2^, expansion to 7 passages yielded 9.24 ± 0.27 × 10^3^ cells/cm^2^ and 2.76 ± 0.49 × 10^3^ cells/cm^2^ from AA treated and Untreated groups at P9, respectively. Experiments were performed in 3 technical replicates. Data are presented as mean ± standard deviation. * *P* < 0.05 and ** *P* < 0.01 compared to Untreated

In current practice, MSCs are commonly harvested at P4 for therapeutic applications because of the loss of viability and increase in senescent cells as well as cell heterogeneity, limiting the potential to produce high yields of clinical grade MSCs. Given that short-term AA supplementation improved chondrogenesis, we tested whether AA supplementation could improve MSC expansion for extended passages. As above, P1 MSCs were thawed and allowed to recover for 1 passage in standard expansion media. We then supplemented expansion media with 1 mM AA (AA) or 0 mM AA (Untreated) at P2 with regular media exchange until P9 (Fig. 4A). MSCs were harvested at P9 and subjected to chondrogenic, osteogenic or adipogenic differentiation (see Methods). We found that extended AA treatment specifically improved chondrogenic potential 4.1-fold relative to Untreated, whereas minimal or no effect was observed for osteogenic or adipogenic potential (Fig. 4B-E). The improvement of extended AA treatment on chondrogenic potential was also supported by higher level of *SOX9* gene expression level (Additional File 1: Figures S7F). We also observed a similar metabolic shift toward OXPHOS, indicated by a significantly higher OCR: ECAR ratio (Fig. 4F) and decreased glucose consumption and lactate production rates (Additional File 1: Figures S7D and S7E).

Cellular senescence and heterogeneity are widely recognized bottlenecks in MSC manufacturing [38, 41]. Similarly, we observed that untreated MSCs with higher passage numbers had cells with increased cell size ranges (Fig. 4H and Additional File 1: Figures S8). In contrast, AA supplementation resulted in reduced heterogeneity in suspended cell diameter at P9 compared to Untreated. Changes in adherent cell morphology between AA-treated and Untreated MSCs at later passage were also measured where Untreated MSCs acquired senescence-like flattened and enlarged morphology while AA-treated MSCs remained spindle-like morphology typical of P4 MSCs (Additional File 1: Figure S7A). MSCs expanded with AA supplementation compared to Untreated MSCs also showed reduced B-gal staining and fewer cells with a characteristic larger, flat cell morphology (Additional File 1: Figure S7B, C). We also noted that AA treated MSCs had a significantly longer mean µMRR T_2_ relaxation time, consistent with the correlation between lower Fe^3+^, a reduced proportion of senescent cells, and improved chondrogenic potential (Fig. 4G).

We next measured the PDT of AA-treated MSCs at each passage up to P9 as above, resulting shorter PDT (3.6 days v. 17.84 days, respectively, at P9) measured throughout expansion, compared to Untreated MSCs (Fig. 4I and Additional File 1: Table S5). Note that the PDT of AA-treated MSCs remained consistent throughout expansion compared to Untreated MSCs indicating stable proliferation rates. Cumulative population doubling level (CPDL) also showed an increase in AA-treated MSCs compared to Untreated MSCs (18.8 vs. 10.5, respectively) (Fig. 4J). We demonstrate that MSCs seeded at 10^5^ cells can yield a nearly 300-fold increase to > 45 billion cells with AA treatment under prolonged expansion conditions compared to 0.14 billion cells in untreated conditions. The difference in proliferation is likely due to the increased proportion of senescent cells and overall decrease in cell health in Untreated controls. Thus, implementation of AA supplementation and monitoring by µMRR as a process control during expansion could provide a simple strategy for improving cell therapy manufacturing of MSCs for specific indications. Taken together, the increased yield, reduced population heterogeneity, and improved chondrogenic capacity observed in our study could fill an unmet need for robust production of clinical-grade MSCs for cartilage repair.

## Discussion

MSCs are an attractive source of chondrocytes for cartilage repair; however, their clinical use is limited by donor-to-donor variation, as well as intra-population heterogeneity and cellular senescence as a result of the lack of standardized MSC expansion protocols [4, 6–8]. Current MSC expansion is often performed with minimal monitoring, posing further challenges in reproducibly generating functionally equivalent MSCs for cell therapy. Thus, identifying adaptive culture conditions that stabilize cell quality while improving chondrogenic potential during MSC manufacturing is critical to overcoming current limitations.

We show that supplementing standard culture media with 1 mM AA one passage prior to differentiation was sufficient to improve the chondrogenic potential of MSCs by 2.6-fold compared to Untreated MSCs (Fig. 1B and C, and Additional File 1: Figure S4). The effect was even stronger (4.1-fold) when AA conditioning was implemented over 7 passages (Fig. 4B). AA supplementation also improved chondrogenic lineage with minimal effects on osteo- and adipo-lineage differentiation. In fact, the osteogenic lineage was significantly suppressed with long-term AA treatment (Figs. 1D-G and 4B-E). AA is an antioxidant, and prior studies found antioxidants and ROS scavengers could decrease cellular senescence [27]. Consistent with this role, we observed a lower percentage of positive beta-gal-stained cells (Additional File 1: Figure S7B), spindle-like morphology (Additional File 1: Figure S7A) and smaller suspended cell diameter (Fig. 4H) compared to Untreated MSCs. Accordingly, AA supplementation had a significant effect on cell yield by maintaining MSCs in a self-renewing state compared to Untreated MSCs in both short and extended AA treatment (Figs. 3A and 4I and J). Thus, supplementing standard MSC culture media with AA during expansion allowed for expansion of a significantly larger number of MSCs with enhanced chondrogenic potential. Although we did not observe evidence of cell death or cytotoxicity, the adaptability to bioreactor conditions as well as evaluation of genetic fidelity of expanded MSCs will be important for adapting to larger scale manufacturing pipelines. Ascorbic acid plays a multifaceted role in influencing cell fate through its antioxidant properties, involvement in collagen synthesis, regulation of epigenetic modifications, and modulation of signalling pathways [42]. Thus, understanding the precise underlying molecular events following AA treatment will be a next step for adapting new expansion approaches to a clinical setting.

Population heterogeneity during expansion also has a negative impact on the therapeutic potential of MSCs [4, 6–8]. Similarly, we observed an increased cellular heterogeneity with passage number as measured by cell size in Untreated MSCs, whereas AA supplementation reduced this heterogeneity (Figs. 3B and 4H). Specifically, AA conditioning resulted in a smaller, consistent average cell size range of 16–18 μm, characteristic of improved chondrogenic potential [14, 15]. AA-treated populations also had a smaller proportion of large MSCs with senescent phenotypes (Figs. 3B and 4H). The therapeutic potential of AA-treated MSCs is supported by prior work showing improved hyaline-like cartilage regeneration in a minipig full-thickness cartilage defect model following implantation of AA and iron treated MSCs [43]. In this study, porcine bone marrow derived MSCs were isolated, expanded, and pre-treated with AA and ferumoxytol or feromoxytol prior to implantation. An improved MR observation of cartilage repair tissue (MOCART) score and MRI demonstrating smooth articular cartilage surface and healthy subchondral bone were observed in the AA and iron treated MSC group, compared to iron alone [43]. The authors suggested that AA could act as a reducing agent for ferric iron (Fe^3+^) to ferrous state (Fe^2+^) [44], which is necessary for catalyzing proline and lysine hydroxylation reactions crucial for collagen formation [45]. These data are consistent with our observation that AA treated human bone marrow derived MSCs showed higher µMRR T_2_ values (decreased Fe^3+^) and improved chondrogenic potential (Figs. 3C and 4G). Together, our work supports the idea that AA supplementation during MSC expansion can enhance cell quality and numbers that could improve chondrogenic repair.

Our work also demonstrates the potential of µMRR for process monitoring of MSC culture for cartilage repair. This tool is particularly attractive, as µMRR is a rapid, label-free method that requires only a small number of cells. Additionally, MSC metabolic profile monitoring could provide another potential CQA to identify MSCs with enhanced chondrogenic potential, given the correlation between chondrogenic potential and OXPHOS activities. Tools including Near-infrared (NIR) and Raman spectroscopy to measure glucose and lactate concentrations in cell culture supernatant [46] are potential metabolic monitoring methods for cell therapy manufacturing given their real-time and non-destructive nature. Given our results showing the benefit of AA priming during MSC expansion combined with correlations between T_2_ values and cellular redox states, we suggest that coupling metabolic manipulation with monitoring and feedback control systems could advance MSC cell manufacturing pipelines for improved clinical outcomes.

## Conclusions

Donor-to-donor variation, intra-population heterogeneity and cellular senescence have impeded the success of MSCs as a standard of care therapy for articular cartilage repair. We demonstrate that AA supplementation during MSC expansion can overcome these bottlenecks and enhance MSC chondrogenic potential. Together, our results suggest that controlling metabolic conditions such as AA supplementation during expansion coupled with process analytical tools, including µMRR could significantly increase the yield and quality of cell therapy products and provide standards for improving the manufacturing pipeline.

## Electronic supplementary material

Below is the link to the electronic supplementary material.

Supplementary Material 1

## Data Availability

All data generated or analyzed during this study are included in this published articles and its supplementary information files.

## Abbreviations

µMRR: Micro-magnetic resonance relaxometry
AA: L-ascorbic acid-2-phosphate
Col2: Type II Collagen
CPDL: Cumulative population doubling level
CQAs: Critical quality attributes
DMEM: D-Glucose Dulbecco’s modified Eagle’s Medium
ECAR: Extracellular acidification rate
FBS: Fetal bovine serum
Gl: Glucose
Gn: Glutamine
ISCT: International Society of Cell and Gene Therapy
MOCART: MR observation of cartilage repair tissue
MSCs: Mesenchymal stromal cells
NIR: Near-infrared
OCR: Oxygen consumption rate
OXPHOS: Oxidative phosphorylation
PAT: Process analytical technology
PBS: Phosphate-buffered saline
PDT: Population doubling time
Py: Pyruvate
ROS: Reactive oxygen species
sGAG: Sulfated glycosaminoglycan
T_2_: Proton transverse relaxation time

## References

[1] Nejadnik H, Hui JH, Feng Choong EP, Tai B-C, Lee EH. Autologous bone marrow–derived mesenchymal stem cells versus autologous chondrocyte implantation: an observational cohort study. Am J Sports Med. 2010;38(6):1110–6. 20392971 10.1177/0363546509359067

[2] Teo AQA, Wong KL, Shen L, Lim JY, Toh WS, Lee EH, et al. Equivalent 10-year outcomes after implantation of autologous bone marrow–derived mesenchymal stem cells versus autologous chondrocyte implantation for chondral defects of the knee. Am J Sports Med. 2019;47(12):2881–7. 31433674 10.1177/0363546519867933

[3] Wakitani S, Nawata M, Tensho K, Okabe T, Machida H, Ohgushi H. Repair of articular cartilage defects in the patello-femoral joint with autologous bone marrow mesenchymal cell transplantation: three case reports involving nine defects in five knees. J Tissue Eng Regen Med. 2007;1(1):74–9. 18038395 10.1002/term.8

[4] Goh D, Yang Y, Lee EH, Hui JHP, Yang Z. Managing the heterogeneity of mesenchymal stem cells for cartilage regenerative therapy: a review. Bioengineering. 2023;10(3):355. 36978745 10.3390/bioengineering10030355 PMC10045936

[5] Maheshwer B, Polce EM, Paul K, Williams BT, Wolfson TS, Yanke A, et al. Regenerative potential of mesenchymal stem cells for the treatment of knee osteoarthritis and chondral defects: a systematic review and meta-analysis. Arthrosc J Arthrosc Relat Surg. 2021;37(1):362–78. 10.1016/j.arthro.2020.05.037 32497658

[6] McLeod CM, Mauck RL. On the origin and impact of mesenchymal stem cell heterogeneity: new insights and emerging tools for single cell analysis. Eur Cell Mater. 2017;34:217. 29076514 10.22203/eCM.v034a14 PMC7735381

[7] Zha K, Li X, Yang Z, Tian G, Sun Z, Sui X, et al. Heterogeneity of mesenchymal stem cells in cartilage regeneration: from characterization to application. Npj Regen Med. 2021;6(1):1–15. 33741999 10.1038/s41536-021-00122-6 PMC7979687

[8] Phinney DG. Functional heterogeneity of mesenchymal stem cells: implications for cell therapy. J Cell Biochem. 2012;113(9):2806–12. 22511358 10.1002/jcb.24166

[9] Volk SW, Wang Y, Hankenson KD. Effects of donor characteristics and ex vivo expansion on canine mesenchymal stem cell properties: implications for MSC-based therapies. Cell Transpl. 2012;21(10):2189–200. 10.3727/096368912X636821 PMC3840229 22472645

[10] Siegel G, Kluba T, Hermanutz-Klein U, Bieback K, Northoff H, Schäfer R. Phenotype, donor age and gender affect function of human bone marrow-derived mesenchymal stromal cells. BMC Med. 2013;11(1):1–20. 23758701 10.1186/1741-7015-11-146 PMC3694028

[11] Herrmann M, Hildebrand M, Menzel U, Fahy N, Alini M, Lang S, et al. Phenotypic characterization of bone marrow mononuclear cells and derived stromal cell populations from human iliac crest, vertebral body and femoral head. Int J Mol Sci. 2019;20(14):3454. 31337109 10.3390/ijms20143454 PMC6678175

[12] Rennerfeldt DA, Raminhos JS, Leff SM, Manning P, Van Vliet KJ. Emergent heterogeneity in putative mesenchymal stem cell colonies: single-cell time lapsed analysis. PLoS ONE. 2019;14(4):e0213452. 30943212 10.1371/journal.pone.0213452 PMC6447157

[13] Russell KC, Phinney DG, Lacey MR, Barrilleaux BL, Meyertholen KE, O’Connor KC. In vitro high-capacity assay to quantify the clonal heterogeneity in trilineage potential of mesenchymal stem cells reveals a complex hierarchy of lineage commitment. Stem Cells. 2010;28(4):788–98. 20127798 10.1002/stem.312

[14] Yin L, Yang Z, Wu Y, Denslin V, Yu CC, Tee CA, et al. Label-free separation of mesenchymal stem cell subpopulations with distinct differentiation potencies and paracrine effects. Biomaterials. 2020;240:119881. 32092592 10.1016/j.biomaterials.2020.119881

[15] Yin L, Wu Y, Yang Z, Tee CA, Denslin V, Lai Z, et al. Microfluidic label-free selection of mesenchymal stem cell subpopulation during culture expansion extends the chondrogenic potential in vitro. Lab Chip. 2018;18(6):878–89. 29459915 10.1039/c7lc01005b

[16] Poon Z, Lee WC, Guan G, Nyan LM, Lim CT, Han J, et al. Bone marrow regeneration promoted by biophysically sorted osteoprogenitors from mesenchymal stromal cells. Stem Cells Transl Med. 2015;4(1):56–65. 25411477 10.5966/sctm.2014-0154 PMC4275011

[17] Yuan X, Logan TM, Ma T. Metabolism in human mesenchymal stromal cells: a missing link between hMSC biomanufacturing and therapy? Front Immunol. 2019;10:977. 31139179 10.3389/fimmu.2019.00977 PMC6518338

[18] Mackay AM, Beck SC, Murphy JM, Barry FP, Chichester CO, Pittenger MF. Chondrogenic differentiation of cultured human mesenchymal stem cells from marrow. Tissue Eng. 1998;4(4):415–28. 9916173 10.1089/ten.1998.4.415

[19] Okita K, Hikiji H, Koga A, Nagai-Yoshioka Y, Yamasaki R, Mitsugi S, et al. Ascorbic acid enhances chondrocyte differentiation of ATDC5 by accelerating insulin receptor signaling. Cell Biol Int. 2023;47(10):1737–48. 37381608 10.1002/cbin.12067

[20] Temu TM, Wu K-Y, Gruppuso PA, Phornphutkul C. The mechanism of ascorbic acid-induced differentiation of ATDC5 chondrogenic cells. Am J Physiol Metab. 2010;299(2):E325–34. 10.1152/ajpendo.00145.2010 PMC2928517 20530736

[21] Sato Y, Mera H, Takahashi D, Majima T, Iwasaki N, Wakitani S, et al. Synergistic effect of ascorbic acid and collagen addition on the increase in type 2 collagen accumulation in cartilage-like MSC sheet. Cytotechnology. 2017;69:405–16. 26572654 10.1007/s10616-015-9924-3 PMC5461231

[22] Yi Y, Wu M, Zhou X, Xiong M, Tan Y, Yu H, et al. Ascorbic acid 2-glucoside preconditioning enhances the ability of bone marrow mesenchymal stem cells in promoting wound healing. Stem Cell Res Ther. 2022;13(1):1–17. 35313962 10.1186/s13287-022-02797-0 PMC8935805

[23] Yu J, Tu Y-K, Tang Y-B, Cheng N-C. Stemness and transdifferentiation of adipose-derived stem cells using L-ascorbic acid 2-phosphate-induced cell sheet formation. Biomaterials. 2014;35(11):3516–26. 24462360 10.1016/j.biomaterials.2014.01.015

[24] Lee S, Lim J, Lee J-H, Ju H, Heo J, Kim Y, et al. Ascorbic acid 2-Glucoside stably promotes the primitiveness of embryonic and mesenchymal stem cells through ten–Eleven translocation-and cAMP-Responsive element-binding protein-1-Dependent mechanisms. Antioxid Redox Signal. 2020;32(1):35–59. 31656084 10.1089/ars.2019.7743

[25] Choi K-M, Seo Y-K, Yoon H-H, Song K-Y, Kwon S-Y, Lee H-S, et al. Effect of ascorbic acid on bone marrow-derived mesenchymal stem cell proliferation and differentiation. J Biosci Bioeng. 2008;105(6):586–94. 18640597 10.1263/jbb.105.586

[26] Traber MG, Stevens JF. Vitamins C and E: beneficial effects from a mechanistic perspective. Free Radic Biol Med. 2011;51(5):1000–13. 21664268 10.1016/j.freeradbiomed.2011.05.017 PMC3156342

[27] Yang M, Teng S, Ma C, Yu Y, Wang P, Yi C. Ascorbic acid inhibits senescence in mesenchymal stem cells through ROS and AKT/mTOR signaling. Cytotechnology. 2018;70:1301–13. 29777434 10.1007/s10616-018-0220-x PMC6214856

[28] Krampera M, Galipeau J, Shi Y, Tarte K, Sensebe L. Immunological characterization of multipotent mesenchymal stromal cells—the International Society for Cellular Therapy (ISCT) working proposal. Cytotherapy. 2013;15(9):1054–61. 23602578 10.1016/j.jcyt.2013.02.010

[29] Thamarath SS, Tee CA, Neo SH, Yang D, Othman R, Boyer LA, et al. Rapid and Live-Cell Detection of Senescence in mesenchymal stem cells by Micro magnetic resonance relaxometry. Stem Cells Transl Med. 2023;12(5):266–80. 36988042 10.1093/stcltm/szad014 PMC10184698

[30] Fujisawa K, Hara K, Takami T, Okada S, Matsumoto T, Yamamoto N, et al. Evaluation of the effects of ascorbic acid on metabolism of human mesenchymal stem cells. Stem Cell Res Ther. 2018;9(1):1–12. 29625581 10.1186/s13287-018-0825-1 PMC5889584

[31] Solchaga LA, Penick KJ, Welter JF. Chondrogenic differentiation of bone marrow-derived mesenchymal stem cells: tips and tricks. Mesenchymal stem cell Assays Appl 2011;253–78. 10.1007/978-1-60761-999-4_20 PMC3106977 21431525

[32] Zhang L, Su P, Xu C, Yang J, Yu W, Huang D. Chondrogenic differentiation of human mesenchymal stem cells: a comparison between micromass and pellet culture systems. Biotechnol Lett. 2010;32:1339–46. 20464452 10.1007/s10529-010-0293-x

[33] Ciuffreda MC, Malpasso G, Musarò P, Turco V, Gnecchi M. Protocols for in vitro differentiation of human mesenchymal stem cells into osteogenic, chondrogenic and adipogenic lineages. Mesenchymal stem Cells Methods Protoc 2016;149–58. 10.1007/978-1-4939-3584-0_8 27236670

[34] Grässel S, Stöckl S, Jenei-Lanzl Z. Isolation, culture, and osteogenic/chondrogenic differentiation of bone marrow-derived mesenchymal stem cells. Somat Stem Cells Methods Protoc. 2012;203–67. 10.1007/978-1-61779-815-3_14 22610563

[35] Hino K, Saito A, Kido M, Kanemoto S, Asada R, Takai T, et al. Master regulator for chondrogenesis, Sox9, regulates transcriptional activation of the endoplasmic reticulum stress transducer BBF2H7/CREB3L2 in chondrocytes. J Biol Chem. 2014;289(20):13810–20. 24711445 10.1074/jbc.M113.543322 PMC4022855

[36] Wehrli BM, Huang W, De Crombrugghe B, Ayala AG, Czerniak B. Sox9, a master regulator of chondrogenesis, distinguishes mesenchymal chondrosarcoma from other small blue round cell tumors. Hum Pathol. 2003;34(3):263–9. 12673561 10.1053/hupa.2003.41

[37] Bonab MM, Alimoghaddam K, Talebian F, Ghaffari SH, Ghavamzadeh A, Nikbin B. Aging of mesenchymal stem cell in vitro. BMC Cell Biol. 2006;7:1–7. 16529651 10.1186/1471-2121-7-14 PMC1435883

[38] Liu J, Ding Y, Liu Z, Liang X. Senescence in mesenchymal stem cells: functional alterations, molecular mechanisms, and rejuvenation strategies. Front Cell Dev Biol. 2020;8:258. 32478063 10.3389/fcell.2020.00258 PMC7232554

[39] Masaldan S, Clatworthy SAS, Gamell C, Meggyesy PM, Rigopoulos A-T, Haupt S, et al. Iron accumulation in senescent cells is coupled with impaired ferritinophagy and inhibition of ferroptosis. Redox Biol. 2018;14:100–15. 28888202 10.1016/j.redox.2017.08.015 PMC5596264

[40] Zhou R-P, Chen Y, Wei X, Yu B, Xiong Z-G, Lu C, et al. Novel insights into ferroptosis: implications for age-related diseases. Theranostics. 2020;10(26):11976. 33204324 10.7150/thno.50663 PMC7667696

[41] Zhang Q, Xu Y, Xu J. Targeting heterogeneity of mesenchymal stem cells. Frontiers in Cell and Developmental Biology. Volume 10. Frontiers Media SA; 2022. p. 894008. 10.3389/fcell.2022.894008 PMC9019297 35465318

[42] Liu TM, Yildirim ED, Li P, Fang HT, Denslin V, Kumar V, et al. Ascorbate and iron are required for the specification and long-term self-renewal of human skeletal mesenchymal stromal cells. Stem cell Rep. 2020;14(2):210–25. 10.1016/j.stemcr.2020.01.002 PMC7013236 32004493

[43] Theruvath AJ, Mahmoud EE, Wu W, Nejadnik H, Kiru L, Liang T, et al. Ascorbic acid and Iron supplement treatment improves stem cell–mediated cartilage regeneration in a Minipig Model. Am J Sports Med. 2021;49(7):1861–70. 33872071 10.1177/03635465211005754 PMC8177720

[44] Timoshnikov VA, Kobzeva TV, Polyakov NE, Kontoghiorghes GJ. Redox interactions of vitamin C and iron: inhibition of the pro-oxidant activity by deferiprone. Int J Mol Sci. 2020;21(11):3967. 32486511 10.3390/ijms21113967 PMC7312906

[45] Rappu P, Salo AM, Myllyharju J, Heino J. Role of prolyl hydroxylation in the molecular interactions of collagens. Essays Biochem. 2019;63(3):325–35. 31350381 10.1042/EBC20180053 PMC6744578

[46] Reyes SJ, Durocher Y, Pham PL, Henry O. Modern sensor tools and techniques for monitoring, controlling, and improving cell culture processes. Processes. 2022;10(2):189.

