# Correction: Metabolic modulation to improve MSC expansion and therapeutic potential for articular cartilage repair

**DOI:** 10.1186/s13287-024-04048-w

**Published:** 2024-11-07

**Authors:** Ching Ann Tee, Daniel Ninio Roxby, Rashidah Othman, Vinitha Denslin, Kiesar Sideeq Bhat, Zheng Yang, Jongyoon Han, Lisa Tucker-Kellogg, Laurie A. Boyer

**Affiliations:** 1 Critical Analytics for Manufacturing Personalised-medicine (CAMP) Interdisciplinary Research Group, Singapore-MIT Alliance for Research and Technology (SMART) Centre, 1 Create Way, Enterprise Wing, #04-13/14, Singapore, 138602 Republic of Singapore; 2 NUS Tissue Engineering Program, Life Sciences Institute, National University of Singapore, 27 Medical Drive, DSO (Kent Ridge) Building, Level 4, Singapore, 117510 Republic of Singapore; 3 Department of Bioresources, University of Kashmir, Hazratbal, Srinagar, 190006 India; 4 Department of Orthopaedic Surgery, National University of Singapore, 1E Kent Ridge Road, NUHS Tower Block 11, Singapore, 119288 Republic of Singapore; 5 Department of Electrical Engineering and Computer Science, Massachusetts Institute of Technology, 50 Vassar St, Cambridge, MA 02139 USA; 6 Department of Biological Engineering, Massachusetts Institute of Technology, 77 Massachusetts Avenue, Cambridge, MA 02139 USA; 7 Cancer and Stem Cell Biology and Centre for Computational Biology, Duke-NUS Medical School, 8 College Rd, Singapore, 169857 Republic of Singapore; 8 Department of Biology, Massachusetts Institute of Technology, 77 Massachusetts Avenue, Cambridge, MA 02139 USA

**Stem Cell Research & Therapy (2024) 15:308**

10.1186/s13287-024-03923-w

The original article has been updated to correct co-author, Kiesar Sideeq Bhat’s name.

