# Supplementary material for: Metabolic modulation to improve MSC expansion and therapeutic potential for articular cartilage repair
Source: Stem Cell Res Ther. 2024 Sep 16;15:308. doi: 10.1186/s13287-024-03923-w (PMC11406821; doi:10.1186/s13287-024-03923-w)
Supplement: Supplementary file 1 — Supplementary Material 1 [file 13287_2024_3923_MOESM1_ESM.docx]

**Supplementary information for**

**Metabolic modulation to improve MSC expansion and therapeutic potential**

**for articular cartilage repair**

Ching Ann Tee, Daniel Ninio Roxby, Rashidah Othman, Vinitha Denslin, Kiseer Sideeq Bhat, Zheng Yang, Jongyoon Han, Lisa Tucker-Kellogg, Laurie A. Boyer

**
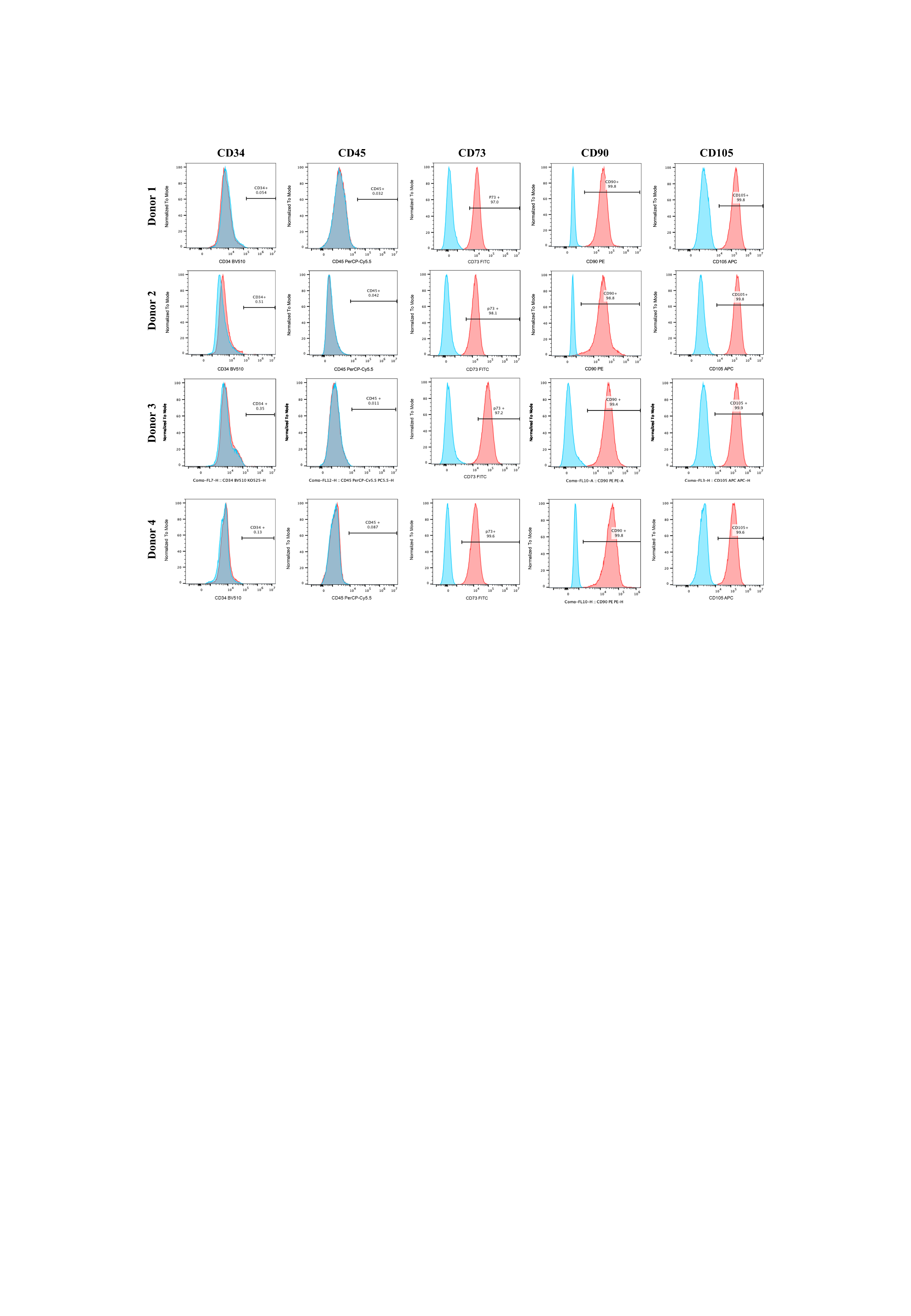
**

**Figure S1: Flow cytometry profiles of MSC surface markers in passage 1 MSCs.**

**
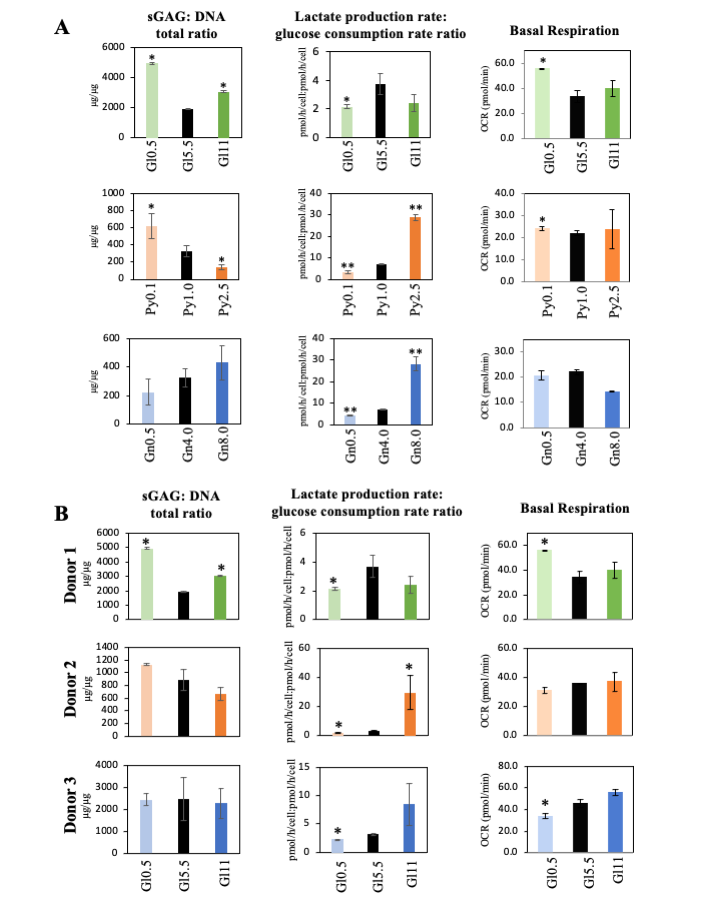
**

**Figure S2. Effect of varying glucose, pyruvate and glutamine concentrations on MSC chondrogenic potential and metabolic profile.** (A) Donor 1 MSCs frozen at P1 were thawed and recovered for 1 passage. At P3 and P4, MSCs were cultured in 10x lower or 2x higher concentrations of standard glucose, pyruvate or glutamine concentrations, namely Gl0.5 (0.5 mM glucose), Gl11 (11 mM glucose), Py0.1 (0.1 mM pyruvate), Py2.5 (2.5 mM pyruvate), Gn0.5 (0.5 mM glutamine), and Gn8.0 (8.0 mM glutamine). MSCs cultured in standard culture media containing 5.5 mM glucose, 1.0 mM pyruvate and 4.0 mM glutamine (Gl5.5/ Py1.0/ Gn4.0) served as Control and highlighted in black bar. At the end of passage 4, MSCs were collected for analysis on chondrogenic potential (the ratio of total sGAG to total DNA) and metabolic profiles (Lactate production rate: glucose consumption rate ratio and oxygen consumption rate, OCR during basal respiration). Lactate production rate to glucose consumption rate ratio was measured by metabolites in spent media with Cedex Bioanalyzer. Oxygen consumption rate (OCR) during basal respiration was measured by Seahorse flux analyzer. (B) Experiments on 10x lower and 2x higher concentrations of standard glucose, Gl0.5 and Gl11, with Gl5.5 served as Control, were repeated on the other 2 donors. At the end of passage 4, MSCs were collected for analysis on chondrogenic potential (Total sGAG: total DNA ratio) and metabolic profiles (Lactate production rate: glucose consumption rate ratio and OCR during basal respiration). Experiments were performed in 3 technical replicates. Data are presented as mean ± standard deviation. * *P* < 0.05 and ** *P* < 0.01 compared to Control (black bar).

**
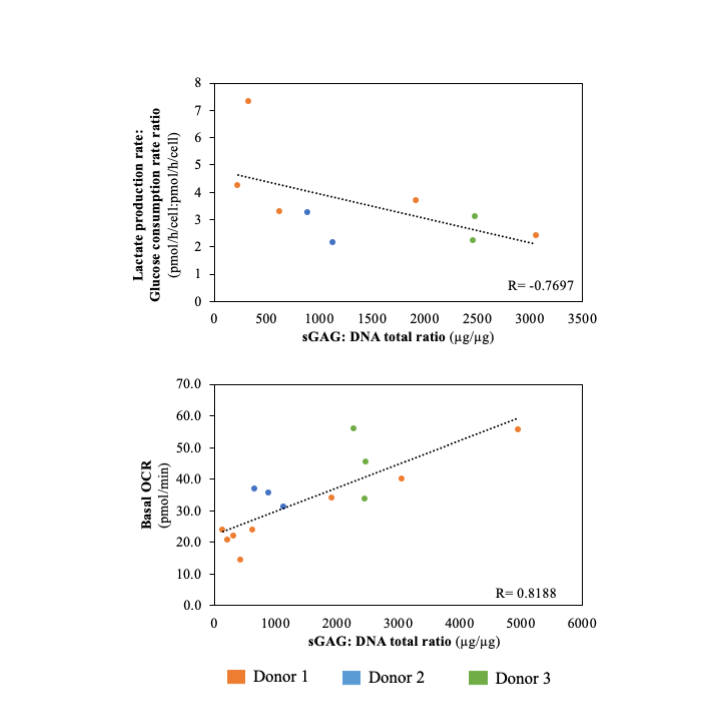
**

**Figure S3. Relationship between MSC chondrogenic potential and metabolic profile.** Correlation analysis between MSC chondrogenic potential indicated by the ratio of total sGAG to total DNA and glycolysis (lactate production rate: glucose consumption rate ratio) or OXPHOS (OCR during basal respiration).


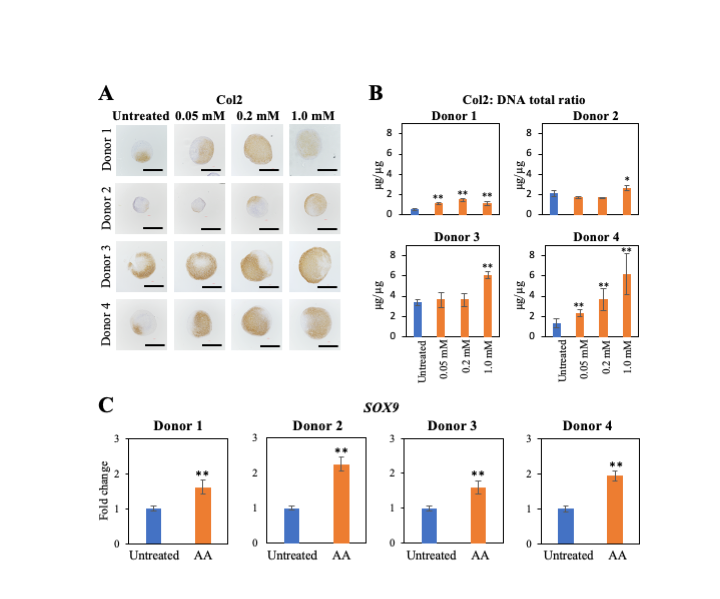


**Figure S4.** **Effect of AA treatment in MSC priming during expansion on subsequent chondrogenic differentiation.** (A) Formation of Col2 indicated by Col2 immunostaining of chondrogenic pellets after 3 weeks of chondrogenic differentiation following 1 passage of 0 (Untreated), 0.05, 0.2 and 1.0 mM AA treatment at P4. 40x magnification; scale bar: 500 µm. Images are representative of 5 replicates per donor. (B) The concentration of Col2 in digested chondrogenic pellets was measured by ELISA and normalized to total DNA per pellet. Experiments were performed with 4 replicates per group. (C) Gene expression level of *SOX9* in MSCs following 1 passage of 0 (Untreated) or 1.0 mM AA treatment at P4. Experiments were performed in 3 technical replicates. Data are presented as mean ± standard deviation. * *P* < 0.05 and ** *P* < 0.01 compared to Untreated.


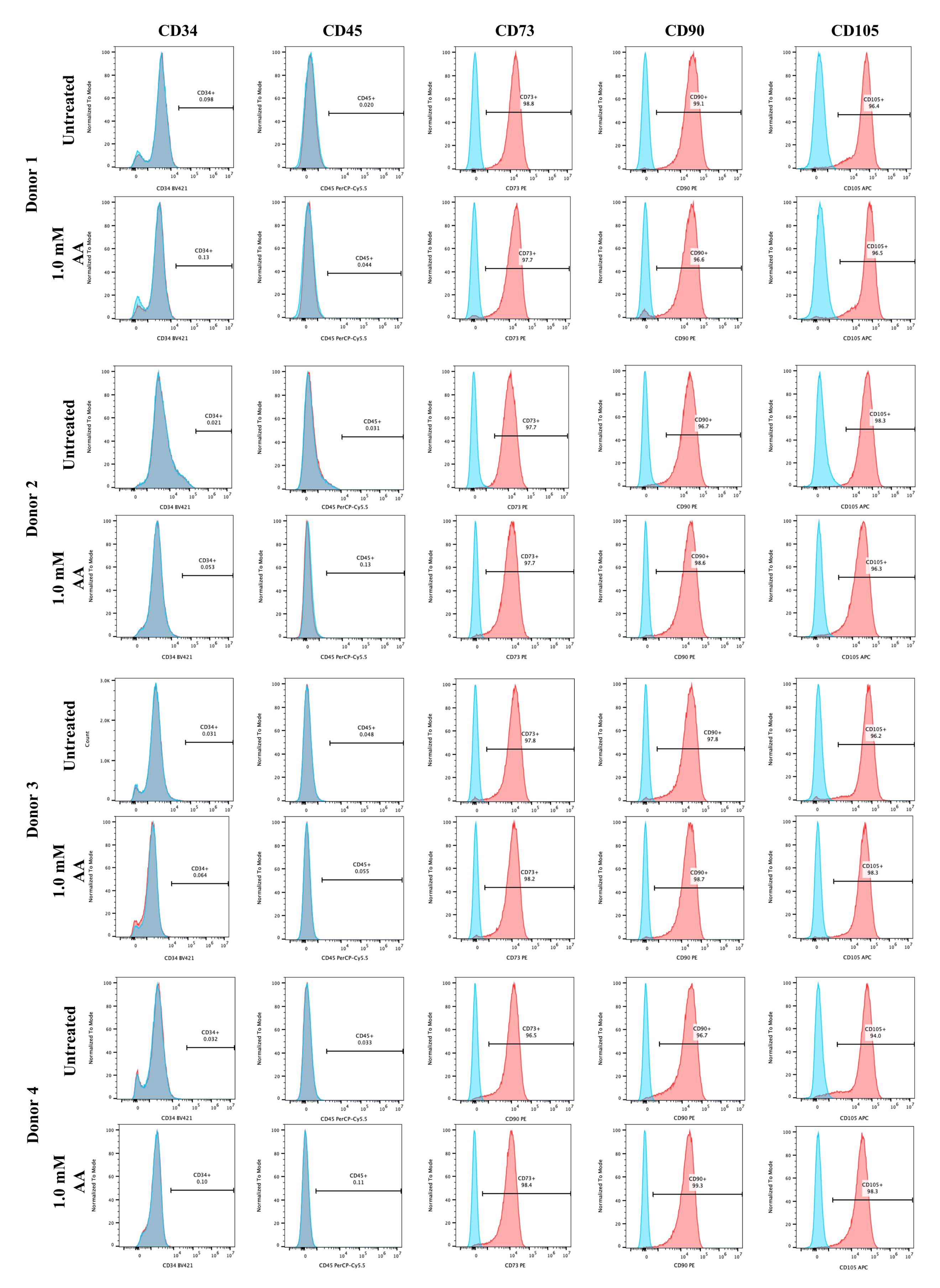


**Figure S5: Flow cytometry characterization of MSC surface markers in Untreated and AA-treated MSCs.**


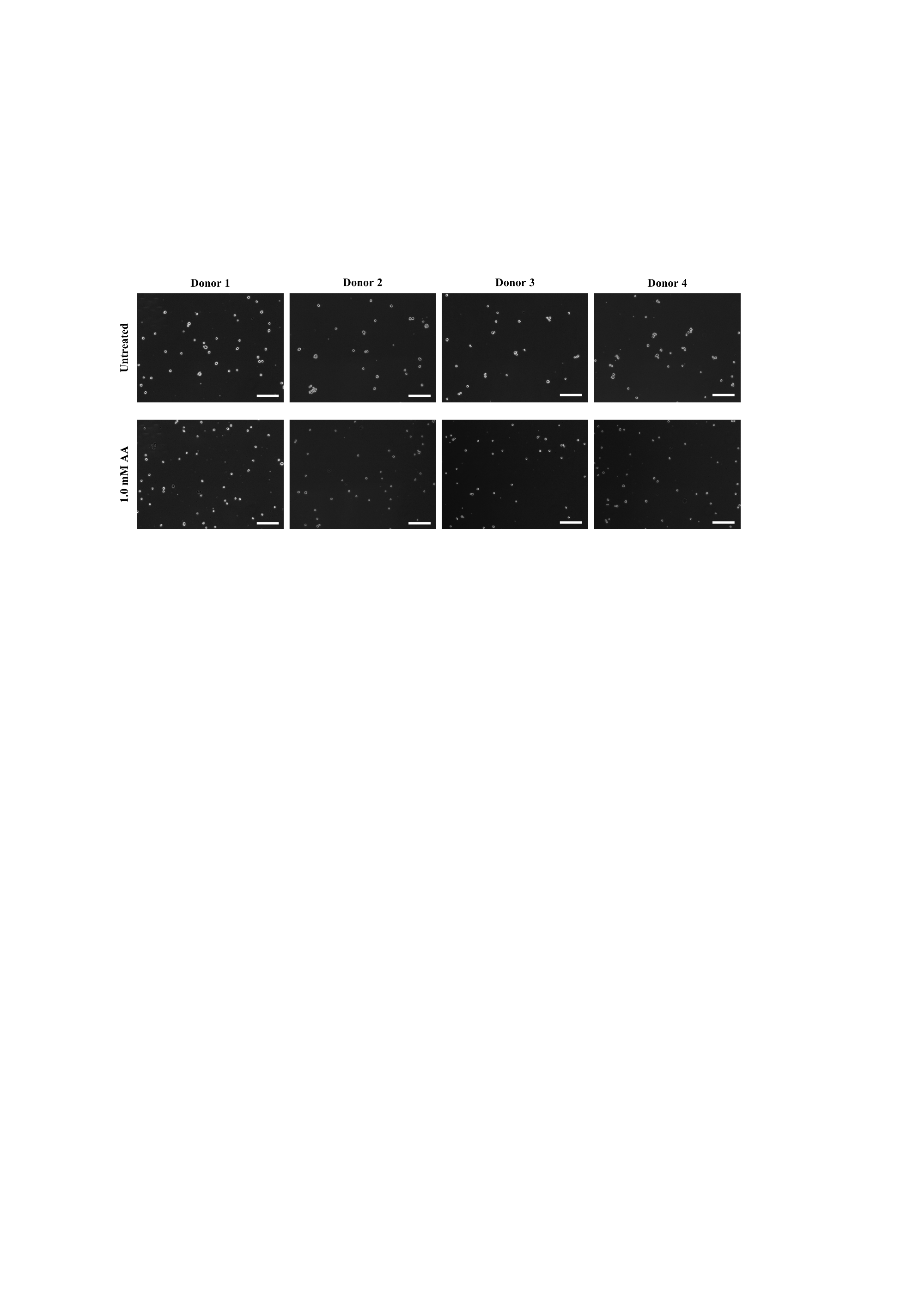


**Figure S6: Effect of AA treatment on MSC proliferation and potential critical quality attributes.** Representative images for suspended cell diameter measurement. Scale bar: 200 µm.


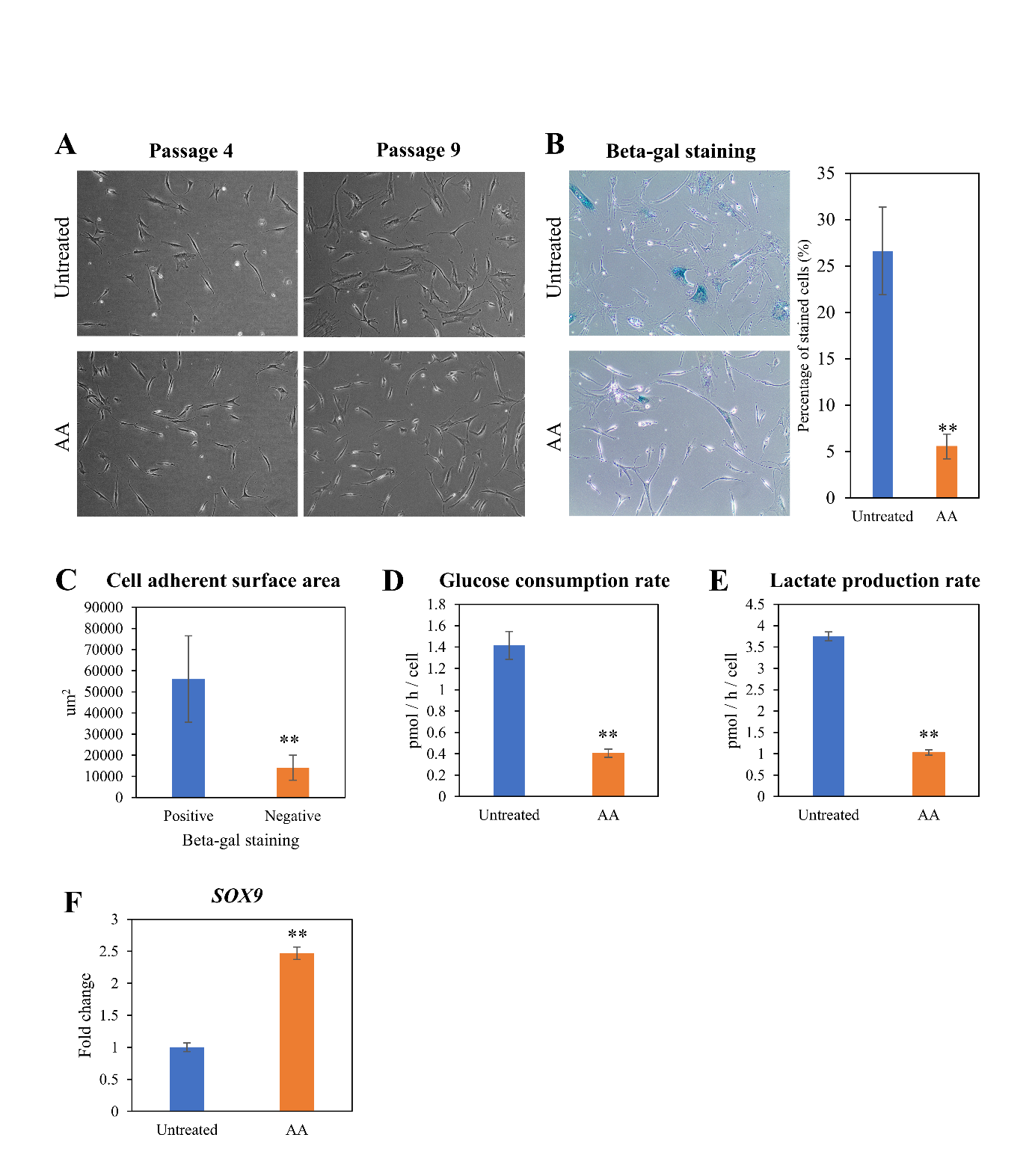


**Figure S7. Effect of long-term AA supplementation in MSC manufacturing.** (A) Morphology of Untreated and AA-treated MSCs at passage 4 and passage 9. (B) Representative images and quantification of beta-gal staining of Untreated or AA-treated MSCs at the end of passage 9. (C) Adherent cell surface area of positively or negatively beta-gal-stained cells quantified with ImageJ. Measurements were calculated from 60-70 cells per group. (D, E) Metabolic profile of Untreated and AA-treated MSCs at the end of passage 9, presented as (D) glucose consumption rate and (E) lactate production rate. (F) Gene expression level of *SOX9* of Untreated or AA-treated MSCs at the end of passage 9. Experiments were performed in 3 technical replicates. Data are presented as mean ± standard deviation. * *P* < 0.05 and ** *P* < 0.01 compared to Untreated.


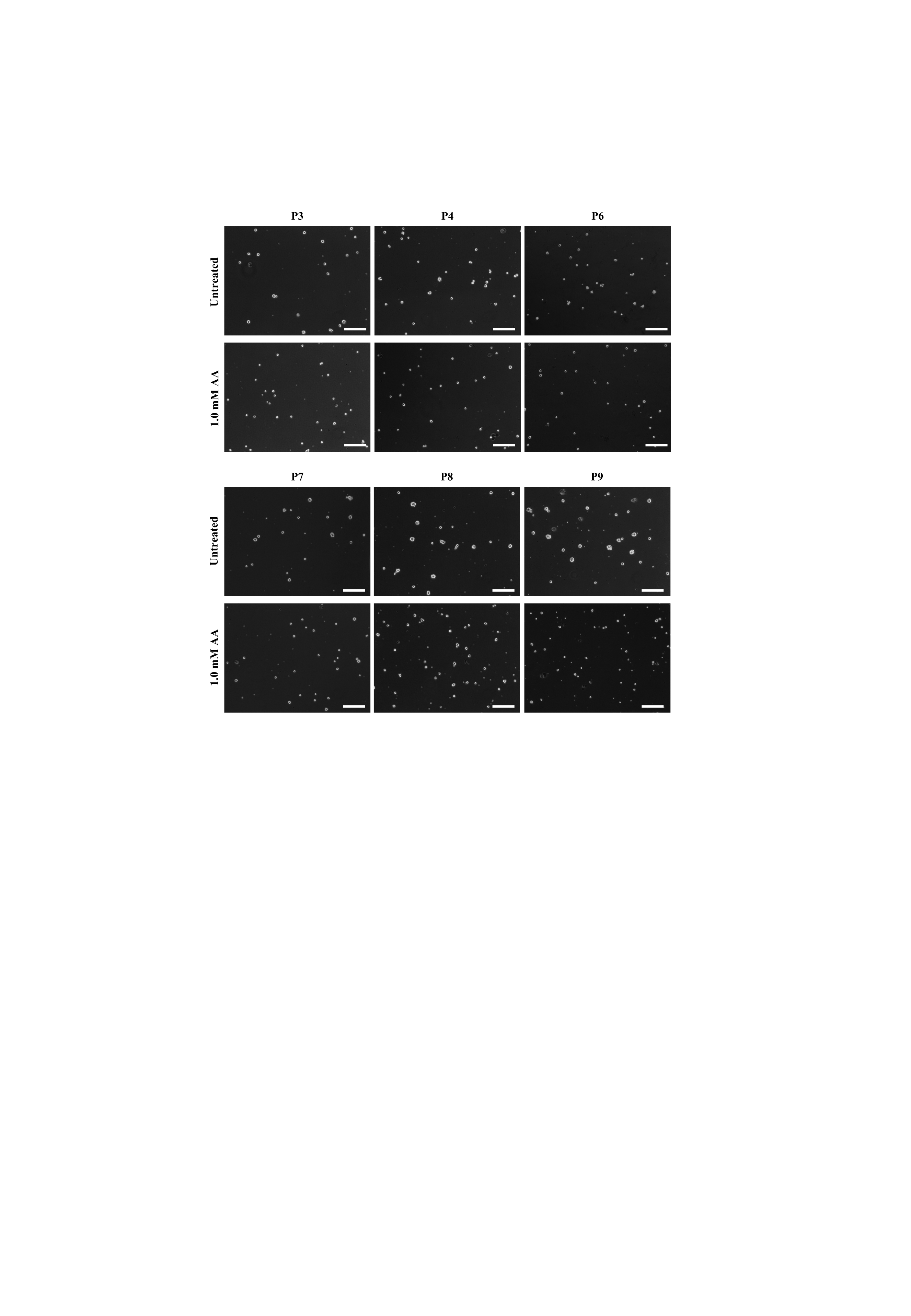


**Figure S8. Effect of long-term AA supplementation in MSC manufacturing.** Representative images for suspended cell diameter measurement. Scale bar: 200 µm.

**Table S1: Description of MSC donors.**

| **Name** | **Source** | **Age** | **Gender** | **Company** |
| --- | --- | --- | --- | --- |
| **Donor 1** | Human Bone Marrow | 25 Y | M | Lonza |
| **Donor 2** | Human Bone Marrow | 19 Y | F | Lonza |
| **Donor 3** | Human Bone Marrow | 37 Y | M | STEMCELL Technologies |
| **Donor 4** | Human Bone Marrow | 24 Y | M | Lonza |

**Table S2: Flow cytometry characterization of MSC surface markers in passage 1 MSCs.**

|  | **% of positive cells** | | | | |
| --- | --- | --- | --- | --- | --- |
| **Donor** | **CD34** | **CD45** | **CD73** | **CD90** | **CD105** |
| **1** | 0.54 | 0.03 | 96.9 | 99.8 | 99.8 |
| **2** | 0.51 | 0.04 | 98.0 | 99.8 | 99.8 |
| **3** | 0.40 | 0.01 | 97.2 | 99.4 | 99.9 |
| **4** | 0.13 | 0.09 | 99.6 | 99.8 | 99.6 |

**Table S3: Flow cytometry characterization of MSC surface markers in Untreated and AA-treated MSCs.**

|  |  | **% of positive cells** | | | | |
| --- | --- | --- | --- | --- | --- | --- |
| **Donor** | **Condition** | **CD34** | **CD45** | **CD73** | **CD90** | **CD105** |
| **1** | **Untreated** | 0.098 | 0.02 | 98.8 | 99.1 | 96.4 |
|  | **1.0 mM AA** | 0.13 | 0.044 | 97.7 | 96.6 | 96.5 |
| **2** | **Untreated** | 0.021 | 0.031 | 97.7 | 96.7 | 98.3 |
|  | **1.0 mM AA** | 0.053 | 0.13 | 97.7 | 98.6 | 96.3 |
| **3** | **Untreated** | 0.031 | 0.048 | 97.8 | 97.8 | 96.2 |
|  | **1.0 mM AA** | 0.064 | 0.055 | 98.2 | 98.7 | 98.3 |
| **4** | **Untreated** | 0.032 | 0.033 | 96.5 | 96.7 | 94 |
|  | **1.0 mM AA** | 0.1 | 0.11 | 98.4 | 99.3 | 98.3 |

**Table S4: Effect of AA treatment on MSC proliferation.** Raw data for cell number on Day 0 during cell seeding and Day 7 after harvesting.

|  |  |  | **Day 0  (cells/ cm^2^)** | **Day 7  (cells/ cm^2^)** |
| --- | --- | --- | --- | --- |
| **Donor 1** | **Untreated** | Reading 1 | 1500 | 10000 |
|  |  | Reading 2 | 1500 | 10286 |
|  |  | Reading 3 | 1500 | 10143 |
|  | **1 mM AA** | Reading 1 | 1500 | 16571 |
|  |  | Reading 2 | 1500 | 15619 |
|  |  | Reading 3 | 1500 | 16095 |
| **Donor 2** | **Untreated** | Reading 1 | 2000 | 5643 |
|  |  | Reading 2 | 2000 | 7643 |
|  |  | Reading 3 | 2000 | 6643 |
|  | **1 mM AA** | Reading 1 | 2000 | 19000 |
|  |  | Reading 2 | 2000 | 17429 |
|  |  | Reading 3 | 2000 | 18214 |
| **Donor 3** | **Untreated** | Reading 1 | 2000 | 8571 |
|  |  | Reading 2 | 2000 | 7619 |
|  |  | Reading 3 | 2000 | 9524 |
|  | **1 mM AA** | Reading 1 | 2000 | 16429 |
|  |  | Reading 2 | 2000 | 15000 |
|  |  | Reading 3 | 2000 | 17857 |
| **Donor 4** | **Untreated** | Reading 1 | 2000 | 13857 |
|  |  | Reading 2 | 2000 | 14429 |
|  |  | Reading 3 | 2000 | 13286 |
|  | **1 mM AA** | Reading 1 | 2000 | 24429 |
|  |  | Reading 2 | 2000 | 23000 |
|  |  | Reading 3 | 2000 | 25000 |

**Table S5: Effect of long-term AA supplementation in MSC manufacturing.** Raw data for cell number on Day 0 during cell seeding and Day 7 after harvesting.

|  |  |  | **Day 0 (cells / cm^2^)** | **Day 7 (cells / cm^2^)** |
| --- | --- | --- | --- | --- |
| **Passage 3** | **Untreated** | Reading 1 | 2000 | 7333 |
|  |  | Reading 2 | 2000 | 7667 |
|  |  | Reading 3 | 2000 | 7500 |
|  | **1 mM AA** | Reading 1 | 2000 | 16833 |
|  |  | Reading 2 | 2000 | 16500 |
|  |  | Reading 3 | 2000 | 16667 |
| **Passage 4** | **Untreated** | Reading 1 | 1500 | 10000 |
|  |  | Reading 2 | 1500 | 10286 |
|  |  | Reading 3 | 1500 | 10143 |
|  | **1 mM AA** | Reading 3 | 1500 | 14143 |
|  |  | Reading 1 | 1500 | 16571 |
|  |  | Reading 2 | 1500 | 15357 |
| **Passage 5** | **Untreated** | Reading 1 | 1500 | 4000 |
|  |  | Reading 2 | 1500 | 3333 |
|  |  | Reading 3 | 1500 | 3667 |
|  | **1 mM AA** | Reading 1 | 1500 | 16667 |
|  |  | Reading 2 | 1500 | 16000 |
|  |  | Reading 3 | 1500 | 16333 |
| **Passage 6** | **Untreated** | Reading 1 | 2000 | 7000 |
|  |  | Reading 2 | 2000 | 7333 |
|  |  | Reading 3 | 2000 | 7167 |
|  | **1 mM AA** | Reading 1 | 2000 | 13500 |
|  |  | Reading 2 | 2000 | 13833 |
|  |  | Reading 3 | 2000 | 13667 |
| **Passage 7** | **Untreated** | Reading 1 | 2000 | 5000 |
|  |  | Reading 2 | 2000 | 4571 |
|  |  | Reading 3 | 2000 | 4786 |
|  | **1 mM AA** | Reading 1 | 2000 | 7714 |
|  |  | Reading 2 | 2000 | 7286 |
|  |  | Reading 3 | 2000 | 7500 |
| **Passage 8** | **Untreated** | Reading 1 | 2000 | 3592 |
|  |  | Reading 2 | 2000 | 3429 |
|  |  | Reading 3 | 2000 | 3510 |
|  | **1 mM AA** | Reading 1 | 2000 | 6857 |
|  |  | Reading 2 | 2000 | 8000 |
|  |  | Reading 3 | 2000 | 7429 |
| **Passage 9** | **Untreated** | Reading 1 | 2000 | 2603 |
|  |  | Reading 2 | 2000 | 2921 |
|  |  | Reading 3 | 2000 | 2762 |
|  | **1 mM AA** | Reading 1 | 2000 | 9429 |
|  |  | Reading 2 | 2000 | 9048 |
|  |  | Reading 3 | 2000 | 9238 |
